# Gut Microbiome and Cardiovascular Diseases

**DOI:** 10.3390/diseases6030056

**Published:** 2018-06-29

**Authors:** Naofumi Yoshida, Tomoya Yamashita, Ken-ichi Hirata

**Affiliations:** Division of Cardiovascular Medicine, Department of Internal Medicine, Kobe University Graduate School of Medicine, Kobe 6500017, Japan; kouchiboy@hotmail.com (N.Y.); hiratak@med.kobe-u.ac.jp (K.H.)

**Keywords:** gut microbiome, cardiovascular diseases, *Bacteroides*

## Abstract

Recent evidence has suggested that the gut microbiome is involved in human health and diseases, such as inflammatory bowel disease, liver cirrhosis, rheumatoid arthritis, and type 2 diabetes. Cardiovascular diseases, which are associated with high morbidity and mortality across the world, are no exception. Increasing evidence has suggested a strong relationship between the gut microbiome and the progression of cardiovascular diseases. We first reported such a relationship with coronary artery disease two years ago. Next-generation sequencing techniques, together with bioinformatics technology, constantly and dramatically expand our knowledge of the complex human gut bacterial ecosystem and reveal the exact role of this bacterial ecosystem in cardiovascular diseases via the functional analysis of the gut microbiome. Such knowledge may pave the way for the development of further diagnostics and therapeutics for prevention and management of cardiovascular diseases. The aim of the current review is to highlight the relationship between the gut microbiome and their metabolites, and the development of cardiovascular diseases by fostering an understanding of recent studies.

## 1. Introduction

The human gastrointestinal tract harbors several hundred trillion bacteria that are collectively referred to as the gut microbiome, which is called the “forgotten organ” because of its important roles beyond digestion and metabolism [1,2]. Growing evidence suggests that the gut microbiome is associated with the pathogenesis of both intestinal and extra-intestinal disorders, such as obesity and other related metabolic diseases, inflammatory bowel disease, and non-alcoholic steatohepatitis, among others [3,4,5]. Next-generation sequencing techniques and multi-omics approaches have constantly and dramatically expanded our knowledge of the microbial world. A new era is dawning with the recognition of the gut microbiome as a “multifunctional organ”. Unsurprisingly, cardiovascular diseases (CVDs) are no exception to this association [6].

CVDs are the leading causes of mortality and morbidity in many developed and developing countries, despite the widespread use of medical therapy in the last decade [7,8,9]. CVDs are responsible for 17.7 million deaths every year (31% of all global deaths), including one of every three deaths in the United States and one of every four deaths in Europe and Japan [8]. By 2030, 40.5% of the US population is projected to have some form of CVD. Between 2010 and 2030, the real, total direct medical costs of CVD are predicted to triple from $273 billion to $818 billion, and the real, indirect costs (owing to lost productivity) for all CVDs are estimated to increase by 61% ($172 billion to $276 billion) [10]. These data strongly support the idea that effective and inexpensive prevention and therapeutic strategies are needed for patients with CVDs. The gut microbiome contributes to human metabolism and the immune system, and is being currently investigated as a diagnostic and therapeutic target for CVDs. Thus, the aim of this review is to discuss the evidence for the relationship between the gut microbiome and CVDs to promote an understanding of the latest perspectives of the role of the gut microbiome in CVDs. Moreover, we have raised several issues that should be considered when interpreting previous evidence.

## 2. Trimethylamine-*N*-oxide and CVDs

A close relationship between the gut microbe-dependent production of trimethylamine-*N*-oxide (TMAO), derived from specific dietary nutrients such as choline and carnitine, and future cardiovascular events has been widely recognized [10]. Trimethylamine (TMA), which is produced by the gut microbial enzymes TMA lyases, is a precursor of TMAO. TMAO can be measured by liquid chromatography-mass spectrometry. Elevated blood TMAO levels have been directly linked to poor outcomes in patients with CVDs, such as coronary artery disease and acute and chronic heart failure (Table 1) [11,12,13,14,15,16]. Tang et al., investigated the relationship between the fasting plasma levels of TMAO and the incidence of major adverse cardiovascular events (death, myocardial infarction, or stroke) during three years of follow-up in 4007 patients undergoing elective cardiac catheterization [13]. They found that the patients in the highest quartile for circulating TMAO levels had a 2.5-fold increased risk of major adverse cardiovascular events, compared with the patients with values in the lowest quartile. Of note, even after adjustment for traditional risk factors, an elevated TMAO level could predict an increased risk of major adverse cardiovascular events [13]. Additionally, high TMAO levels were observed in patients with stable heart failure compared to healthy subjects [11]. This result suggests that the gut microbiome may play a role in the development and progression of heart failure. They also showed that elevated TMAO levels were associated with a 2.2-fold increase in the risk of mortality, after an adjustment for traditional risk factors and the brain natriuretic peptide. Moreover, the blood TMAO levels were associated with coronary plaque vulnerability, as assessed by optical coherence tomography, and the long-term risks of cardiovascular events in patients with acute coronary syndrome [14]. The latest metagenome-wide association study demonstrated the microbial characterization of coronary artery disease (CAD) patients and showed that the gut microbial enzymes that produce TMA were enriched in the patients with CAD compared to the healthy controls [17].

As different gut microbial compositions generate different levels of TMAO [18], higher blood TMAO levels and an increased CVD risk can be attributed to a TMA-producing microbiome harboring TMA lyases. These findings support the idea that prevention of CVD is feasible through gut microbial modulation. However, the area under the receiver operating characteristic curve, based on TMA lyases, was not sufficient to predict the incidence of CAD (Area Under the Curve = 0.63). Moreover, a recent clinical trial has shown that fish consumption increases the circulating TMAO levels, highlighting the substantial limitations in our current understanding of the relationship between diet and gut microbial TMAO production [18]. Moreover, all available clinical studies are cross-sectional studies or cohort studies, not interventional studies. Further research is needed to elucidate whether TMAO contributes directly to the progression of CVD or reflects the presence of a deleterious colonic microbial metabolism, dietary habits, or renal tubular dysfunction. In addition, the distribution of TMAO levels in the general population is unknown, and standard reference values are not currently available [19]. A detailed understanding of the biological role of TMAO in CVD patients is crucial for evaluating the feasibility of developing drugs that affect the TMAO levels or the possibility of using TMAO as a marker of CVD.

## 3. Other Gut Microbial Metabolites and CVD

There are a number of other gut microbial metabolites in addition to TMA. These metabolites have also been reported to have a link to CVDs. Indoxyl sulfate is produced by gut microbial tryptophanases that convert dietary tryptophan into indole, which is then converted to indoxyl and indoxyl sulfate in the liver by the sequential actions of cytochrome P450 enzymes and sulfotransferase 1A1. Indoxyl sulfate has been shown to have pro-inflammatory and pro-oxidant effects in cardiomyocytes and cardiac fibroblasts. Furthermore, recent reports have shown that indole and indoxyl sulfate affect the arterial blood pressure via peripheral and central mechanisms that depend on serotonin signaling in rats [20].

Short chain fatty acids (SCFAs), produced by the colonic bacterial fermentation of dietary fiber, contribute a significant proportion of the daily energy requirement [21]. SCFAs, especially butyrate and propionate, play an important role in regulatory T cell differentiation and intestinal tract immune regulation. The increased production of acetate by the gut microbiota of rodents leads to the activation of the parasympathetic nervous system, which promotes increased glucose-stimulated insulin secretion, hyperphagia, and obesity. However, no reports have described the direct impact of SCFAs on the incidence and progression of cardiovascular diseases [22].

The gut microbiome utilizes sulfur-containing compounds to produce hydrogen sulfide. Hydrogen sulfide is an important biological mediator that is involved in various physiological processes, including the regulation of arterial blood pressure [23]. Moreover, phenylacetylglutamine is a product that is formed by the conjugation of phenylacetate and glutamine. High serum levels of phenylacetylglutamine have been observed in patients with advanced chronic kidney disease, and as a strong and independent risk factor for overall mortality and cardiovascular diseases [24]. P-cresyl sulfate, a secondary metabolism of p-cresol, is also a microbial metabolite. Increased levels of p-cresyl sulfate are associated with worse outcomes in patients with chronic kidney disease [25].

These results suggest that gut microbial metabolites may play an important role in the development of CVD. Further studies are warranted to elucidate the causal relationship between these metabolites and CVD.

## 4. Alterations of the Gut Microbial Structure Associated with CVD

Several studies have been conducted to elucidate which gut bacterial species are involved in the incidence and progression of CVD (Table 2) [17,26,27,28]. We were the first to report that the incidence of CAD was linked to an alteration of the gut microbial composition [28,29]. We have reported a lower abundance of the phylum *Bacteroidetes* and a higher abundance of the order *Lactobacillales* in patients with CAD compared to non-CAD patients with coronary risk factors, such as diabetes, hypertension, or dyslipidemia, and healthy volunteers using terminal restriction fragment length polymorphism analysis, which is one of the most well-established and reliable 16S rRNA-based methods. The *Firmicutes/Bacteroidetes* ratio, an indicator of dysbiosis, increased in the CAD patients compared with the non-CAD controls. Interestingly, our data revealed that the CAD patients were significantly more likely to be categorized as enterotype Ш, which is characterized by low levels of *Bacteroides*, compared with the non-CAD controls. Last year, a metagenome-wide association study of fecal samples from 218 CAD patients and 187 healthy subjects from China was reported [17]. The abundance of *Enterobacteriaceae* was significantly higher in the CAD patients compared to the healthy subjects. The abundance of *Streptococcus* spp. was also significantly higher in the patients with CAD than in the healthy subjects. This may be due to the use of proton pump inhibitors in CAD patients [30]. Consistent with our results, *Bacteroides* spp. were significantly depleted in the CAD patients. Given that *Bacteroides* spp. are known to have an important role in maintaining a healthy gut ecosystem [31], and that the abundance of *Bacteroides* spp. was found to decrease in patients with atherosclerotic ischemic stroke and transient ischemic attack [27], *Bacteroides* spp. may have the potential to regulate atherosclerosis progression. Furthermore, *Faecalibacterium prausnitzii*, which exhibits anti-inflammatory effects [32], was also significantly depleted in the CAD patients. Of note, the co-abundance network structure differed between the two groups. The negative correlations between *Streptococcus* spp. and *Bacteroides* spp. were observed only in the CAD patients. On the other hand, the positive correlation between *Bacteroides* spp. and *Erysipelotrichaceae* bacterium was seen only in the healthy subjects. These results implied that a peculiar inter-species relationship in the gut microbiome may exist in CAD patients compared to healthy subjects.

Additionally, there are some studies that have demonstrated the relationship between the gut microbiome and heart failure (HF). Kamo et al., first reported the gut microbial difference in Japanese heart failure patients [33]. They performed a 16S rRNA gene sequencing analysis of fecal samples from 12 HF patients and 12 age-matched healthy subjects. They further compared the gut microbiome in HF patients according to age; the gut microbiome in the 12 HF patients younger than 60 years of age were compared with those of the 10 HF patients 60 years of age or older. Although the richness and diversity of the gut microbiota were not significantly different between the HF patients and healthy subjects, *Dorea* and *Clostridium* were less abundant in the HF patients than in the healthy subjects. Moreover, older HF patients had a lower abundance of *Bacteroidetes* and a higher abundance of *Proteobacteria* compared to the younger HF patients. There is also a report from China that shows a metagenomic analysis of fecal samples from patients with chronic HF [34]. They enrolled 53 HF patients and 41 controls with risk factors and compared the compositions of their gut microbiomes. *Ruminococcus*, *Acinetobacter*, and *Veillonella* increased in the HF patients, whereas *Alistipes*, *Faecalibacterium*, and *Oscillibacter* decreased. In line with the previous report, *Faecalibacterium prausnitzii* decreased in the HF patients compared to the controls. The results of these studies suggest that an altered gut microbiome may have an impact on the development and progression of heart failure. This evidence paves the way for further studies investigating the gut microbiome in the prevention and management of CVD.

## 5. Alternations in Gut Microbial Function Associated with CVD

In addition to the compositional characteristics, the functional characteristics of the gut microbiome have been investigated in order to delineate the mechanisms related to the development of CVD. Although metagenomic shotgun sequencing analysis is the main method to examine the functional characteristics, methods are being developed to predict functional profiles from taxonomic profiles. Phylogenetic Investigation of Communities by Reconstruction of Unobserved States (PICRUSt) is a bioinformatics software package designed to predict metagenomic functional content from the 16S rRNA gene [35]. The Kyoto Encyclopedia of Genes and Genomes (KEGG) modules are usually used to construct a functional map of the gut microbiome [36].

The first shotgun sequencing of the gut metagenome in patients with symptomatic atherosclerotic plaques in their carotid arteries was a study with a small number of samples [27]. They showed that genes that encode proteins involved in peptidoglycan synthesis were enriched, and those that encode phytoene dehydrogenases were depleted in the patients compared to these genes in healthy subjects. Considering that gut bacterial function differs even within the same strain, a metagenomic shotgun sequencing study must provide us with further detailed information. Five years later, Jie et al., have reported a metagenomic shotgun sequencing study with 218 CAD patients and 187 healthy subjects [30]. They revealed alterations in gut microbial functional modules in CAD patients, such as the phosphotransferase system, amino acid transporters, vitamin metabolism, lipopolysaccharide biosynthesis, and the activities of SCFAs and TMA lyases.

With regard to HF, Cui et al., have investigated the metabolic patterns of the gut microbiome in patients with chronic HF to provide direct evidence and a comprehensive understanding of gut microbial dysbiosis [34]. Fifty-three chronic HF patients (ischemic cardiomyopathy, *n* = 29; dilated cardiomyopathy, *n* = 24) and 41 controls with risk factors were enrolled. They found an elevation in the microbial genes for lipopolysaccharide biosynthesis, tryptophan, and TMAO generation in the chronic HF patients. This result provides a convincing explanation for the increased plasma lipopolysaccharide levels in HF patients [37], because the main source of lipopolysaccharides is the gut/gut microbiome. Moreover, increased expression of the genes for phosphotransferase systems and decreased gene expression for the synthesis and transport of amino acids, nucleotide sugar biosynthesis, and the iron transport system were observed in the HF patients compared with the controls. These disease-dependent unique features in the functional capacity may give us clues for novel therapeutic approaches.

## 6. Issues to Be Considered When Interpreting the Studies

Most clinical studies compare the gut microbial composition between patients and healthy controls. Administration of medication has a substantial effect on the gut microbiome, and medication-matched controls are required to elucidate the impact of the gut microbiome on disease progression. Moreover, the studies mentioned above have provided useful characterization of the fecal microbial profile in patients with CVD; however, we are still struggling with these descriptive data. A specific gut microbiome-based target to prevent CVD has yet to emerge, which is the greatest challenge that we are currently facing. It may take a little more time to conduct a large cohort study or a translational study to promote a deeper understanding of how the gut microbiome directly contributes to CVD. While we already know that diet, prebiotics, probiotics, a specific IgA antibody, and enzymes can modulate the gut microbiome and its function [38,39], these interventions for patients with CVD are constrained by ethical considerations or funding limitations. In such cases, an in vitro fermentation system simulating the human intestinal tract may help to evaluate the functionality or safety of these interventions under highly reproducible conditions without the ethical issues [40]. Specifically, we can culture feces from patients with prebiotics or probiotics in an in vitro fermentation system and analyze how the gut microbiome, and its metabolites and functions, are changed after the intervention. Of note, we have observed some discrepancies between the findings in humans and mice. These may be due to the differences in the natural gut microbiome. It is important to pay attention to the complexities of translating the findings from an animal model to humans.

## 7. Conclusions

In summary, recent evidence on the potential interaction between the gut microbiome and cardiovascular diseases is intriguing. With increasing awareness of the relationship between the gut microbiome and CVD, we have high expectations for the clinical application of gut microbiome modulation. Further studies, focusing on a more specific and mechanistic understanding of the gut microbiome in the pathogenesis of CVD, are necessary to develop novel diagnostic and therapeutic strategies for CVD.

## Figures and Tables

**Table 1:** Major clinical reports demonstrating the impact of circulating trimethylamine-*N*-oxide (TMAO) levels on cardiovascular diseases (CVD).

Year	Study Population	Number of Subjects	Main Outcome	Follow-Up Period	Results
2013 *N. Engl. J. Med.*	Patients who were undergoing elective diagnostic cardiac catheterization	4007 in USA	Major cardiovascular events (myocardial infarction, stroke), or death	3 years	Increased TMAO levels were associated with an increased risk of major adverse cardiovascular events or death
2014 *J. Am. Coll. Cardiol.*	Stable heart failure patients underwent elective coronary angiographic evaluation	720 in USA	All-cause mortality (death)	5 years	Elevated TMAO levels portended higher long-term mortality risk
2015 *J. Card Fail*	Chronic systolic heart failure with comprehensive echocardiographic evaluation	112 in USA	Adverse clinical events (death/transplantation)	5 years	Higher TMAO levels were associated with a higher incidence of death/transplantation
2016 *Heart*	Acute heart failure	972 in UK	All-cause mortality (death) and a composite of death or re-hospitalization due to heart failure (death/HF)	1 year	Elevated levels were associated with a higher incidence of death/HF
2016 *Am. J. Cardiol.*	Coronary artery disease	26 in China	Coronary plaque vulnerability assessed by optical coherence tomography	-	Plasma TMAO level was significantly higher in patients with plaque rupture than in those without plaque rupture
2017 *Clin. Chem.*	Acute myocardial infarction	1079 in UK	Composite of all-cause mortality and re-infarction (death/myocardial infarction)	2 years	TMAO levels were associated with death/MI

**Table 2:** Clinical reports demonstrating the gut microbiome in patients with CVD.

Year	Study Population	Country	Analysis	Results
2012 *Nat. Commun.*	12 patients with symptomatic atherosclerosis (myocardial infarction or cerebrovascular events) and 13 age- and sex-matched healthy individuals.	Sweden	Gut metagenome	*Collinsella* ↑, *Eubacterium* ↓, *Roseburia* ↓ in patients with symptomatic atherosclerosis.
2015 *J. Am. Heart Assoc.*	141 patients with stroke and transient ischemic attack (stroke/TIA patients) and 94 asymptomatic controls.	China	16S rRNA V4 region	*Enterobacteriaceae* ↑, *Proteobacteria* ↑, *Escherichia/Shigella* ↑, *Bacteroidetes* ↓, *Bacteroidales* ↓, *Bacteroidaceae* ↓, *Bacteroides* ↓ in stroke/TIA patients.
2016 *J. Atheroscler. Thromb.*	39 coronary artery disease (CAD) patients, 30 age- and sex-matched no-CAD controls with coronary risk factors, and 50 healthy volunteers without coronary risk factors.	Japan	Terminal restriction fragment length polymorphism	*Firmicutes/Bacteroidetes* ratio ↑, *Lactobacillales* ↑, *Bacteroides* + *Prevotella* ↓ in CAD.
2017 *Nat. Commun.*	218 individuals with atherosclerotic cardiovascular disease (ACVD) and 187 healthy controls.	China	Gut metagenome	*Enterobacteriaceae* (*Escherichia coli, Klebsiella* spp., and *Enterobacter aerogenes*), *Streptococcus* spp., *Lactobacillus salivarius*, *Solobacterium moorei*, *Atopobium parvulum, Ruminococcus gnavus, Eggerthella lenta* ↑, *Roseburia intestinalis* ↓, *Faecalibacterium* cf. *prausnitzii* ↓, *Bacteroides* spp. ↓, *Prevotella copri* ↓, *Alistipes shahii* ↓ in ACVD.
